# Supplementary material for: Population genetic structure and variability in Lindera glauca (Lauraceae) indicates low levels of genetic diversity and skewed sex ratios in natural populations in mainland China
Source: PeerJ. 2020 Jan 3;8:e8304. doi: 10.7717/peerj.8304 (PMC6944114; doi:10.7717/peerj.8304)
Supplement: Table S1 [file peerj-08-8304-s001.doc]

**Supplementary Tables**

**Table S1.** **Genetic characteristics of 13 nuclear SSR loci in 22 populations of *Lindera glauca* (*N* = 300).**

| **Locus** | **Primer sequence (5′-3′)** | **Repeat Motif** | **Size (bp)** | ***Na*** | ***Ho*** | ***He*** | **PIC** | ***A_R_*** | **GenBank accession no.** |
| --- | --- | --- | --- | --- | --- | --- | --- | --- | --- |
| P-287 | F: ACTCTATGTAGCAAGGGCGG R: AGAGAAGGCAGGGTTCTGGT | (AGC)5 | 162 | 7 | 0.390 | 0.735 | 0.688 | 2.702* | MF163041 |
| P-294 | F: TCCATCCCTAATGTCCTCTAGC R: GCAGTGGTTAAACACCTTCAAA | (AG)8 | 180 | 5 | 0.241 | 0.478 | 0.442 | 2.007* | MF163042 |
| P-298 | F: GCAACAGATGCAAAGCAGTC R: GGATAGCATTCAGGAGCGAA | (AG)7 | 194 | 3 | 0.244 | 0.428 | 0.386 | 1.866* | MF163043 |
| XBLG-036 | F: CATCACCTCCCTCAAATCCC R: GTTTCCGAAATTCTCGAGGC | (AG)7 | 263 | 6 | 0.210 | 0.495 | 0.444 | 2.020* | KX545438 |
| XBLG-055 | F: CCTCTTCAAACCAAACCTCC R: CTGCAACTCCATGTGAGGG | (AAG)5 | 236 | 6 | 0.424 | 0.754 | 0.711 | 2.774* | KX545442 |
| XBLG-058 | F: AGTCCAGGCTAACCAGACTCC R: CCCAGTTTGCCAGGTAAGAA | (AAC)6 | 277 | 7 | 0.553 | 0.637 | 0.594 | 2.431* | KX545444 |
| XBLG-060 | F: ATTCCACCCATTCCCTTCTT R: GATTCTAAGAAGAAGAAGAAAGTACCC | (AAG)6 | 197 | 8 | 0.292 | 0.691 | 0.641 | 2.569* | KX545445 |
| XBLG-062 | F: AACATCATTCCCTCCATCCA R: CCAGCCAGTTAGGGTTTCAC | (AATCC)5 | 192 | 5 | 0.532 | 0.655 | 0.600 | 2.439* | KX545446 |
| XBLG-063 | F: CATGGCAACGCAAATCCTAT R: CTAGATCCTTTGGCCATGTTT | (ATC)6 | 196 | 6 | 0.224 | 0.380 | 0.363 | 1.807* | KX545447 |
| XBLG-076 | F: GGATGCTCTAAGGTGCTTGC R: GGAATCGCCATTCTCCCT | (AG)7 | 182 | 7 | 0.451 | 0.739 | 0.694 | 2.722* | KX545450 |
| XBLG-083 | F: CTCTCTCATCGATCCACCG R: AAACCCAACACTGTACAACCTAAA | (AAG)5 | 186 | 4 | 0.563 | 0.620 | 0.545 | 2.295* | KX545452 |
| XBLG-089 | F: TGTCTTGTGATCGAAATCAGG R: ACTTCAGAGGCATTCCAGCA | (AG)7 | 177 | 5 | 0.403 | 0.569 | 0.540 | 2.268* | KX545455 |
| XBLG-097 | F: TTTGGGAAAGTCCCATGAAA R: GGGTACAAGTGGATACAATGAGG | (ATC)6 | 193 | 5 | 0.410 | 0.642 | 0.574 | 2.375* | KX545457 |
| Mean |  |  |  | 5.7 | 0.380 | 0.602 | 0.556 | 2.329 |  |

*Notes*: *Na* = number of alleles at nSSR loci; *Ho* = observed heterozygosity; *He* = expected heterozygosity; *A_R_* = allelic richness; PIC = polymorphism information content; * = significant deviations from HWE determined for each locus in the population using GENEPOP (P < 0.005).
