# Supplementary material for: Population genetic structure and variability in Lindera glauca (Lauraceae) indicates low levels of genetic diversity and skewed sex ratios in natural populations in mainland China
Source: PeerJ. 2020 Jan 3;8:e8304. doi: 10.7717/peerj.8304 (PMC6944114; doi:10.7717/peerj.8304)
Supplement: Table S2 [file peerj-08-8304-s002.doc]

***Supplementary Material***

**Table S2.** **Genetic characteristics of five chloroplast SSR maerkers and results of genotyping in *L. glauca* (*N* = 300).**

| **Locus** | **Primer sequence (5′-3′)** | **Repeat Motif** | **Size (bp)** | ***Na*** | ***Dv*** | **PIC** | **GenBank accession no.** |
| --- | --- | --- | --- | --- | --- | --- | --- |
| CPLG01 | F: CTCACCCTTCGTTGAACCAT R: GGCAGAATTTTGTTTCCAGG | (AT)6 | 221 | 3 | 0.185 | 0.171 | MF188124* |
| CPLG02 | F: TCCAACGGAATCCCACTTAC R: CCATAAATCCCGAGATGGAA | (TC)6(T)10 | 381 | 2 | 0.115 | 0.108 | MF188124* |
| CPLG03 | F: ACGATGACTTTGGTTTTCGC R: AGAAAGACCCGCCTGTCATA | (TC)7 | 207 | 2 | 0.249 | 0.218 | MF188124* |
| CPLG04 | F: GGGAAAAGACCCGTATCCAT R: GGATCGGATCGAATTGAAAA | (GA)6 | 244 | 3 | 0.164 | 0.155 | MF188124* |
| CPLG05 | F: TTGTACTGATTGGGGGCTTC R: GGGGTTCTTAAGCTTTTCGATT | (AT)10 | 190 | 3 | 0.176 | 0.168 | MF188124* |

*Notes*: Size = expected length of the PCR product in base pairs; *Na* = number of alleles per locus at cpSSR loci; *Dv* = observed genetic diversity for each locus; PIC = polymorphism information content; * = primer pair designed according to the chloroplast genome (Xiong et al., 2016).
