# Supplementary material for: Population genetic structure and variability in Lindera glauca (Lauraceae) indicates low levels of genetic diversity and skewed sex ratios in natural populations in mainland China
Source: PeerJ. 2020 Jan 3;8:e8304. doi: 10.7717/peerj.8304 (PMC6944114; doi:10.7717/peerj.8304)
Supplement: Table S3 [file peerj-08-8304-s003.doc]

**Table S3.** **List of haplotypes detected at five cpSSR loci in 22 populations of *L. glauca*.** Private haplotypes are highlighted in red, and their corresponding frequencies are shown in the last column.

|  | **Wild populations** | | | | | | | | | | | | | | | | | | | | **Cultivated populations** | |  |
| --- | --- | --- | --- | --- | --- | --- | --- | --- | --- | --- | --- | --- | --- | --- | --- | --- | --- | --- | --- | --- | --- | --- | --- |
| **Haplotype** | **A**  **T**  **M** | **J**  **G**  **S** | **L**  **D**  **Z** | **S**  **J**  **G** | **N**  **T**  **B** | **Y**  **T**  **H** | **D**  **B**  **S** | **H**  **M**  **F** | **T**  **M**  **S** | **S**  **Q**  **S** | **L**  **Y**  **S** | **K**  **Y**  **S** | **F**  **J**  **S** | **W**  **Y**  **S** | **Z**  **J**  **S** | **W**  **J**  **S** | **G**  **J**  **S** | **N**  **H**  **S** | **F**  **H**  **S** | **Z**  **J**  **J** | **S**  **Z**  **Y** | **H**  **Z**  **Y** | **Frequencies of private haplotypes** |
| **H1** |  | X |  |  |  |  |  |  |  |  |  |  |  |  |  |  |  |  |  |  |  |  |  |
| **H2** |  | **X** |  |  |  |  |  |  |  |  |  |  |  |  |  |  |  |  |  |  |  |  | 0.0333 |
| **H3** |  |  |  |  |  |  |  |  |  |  |  |  |  |  |  |  |  |  |  |  | X | X |  |
| **H4** |  |  |  |  |  |  |  |  |  |  |  |  | X |  |  |  |  |  |  | X |  |  |  |
| **H5** |  |  |  |  |  |  |  |  |  |  |  |  | X |  |  |  |  |  |  | X |  |  |  |
| **H6** |  |  |  |  |  |  |  |  |  |  |  |  |  |  |  |  |  |  |  |  | **X** |  | 0.5000 |
| **H7** |  |  |  |  |  |  |  |  |  |  |  |  | X |  |  |  |  |  | X | X |  |  |  |
| **H8** |  |  |  |  |  |  |  |  |  |  |  |  |  |  |  |  |  |  |  | **X** |  |  | 0.0333 |
| **H9** |  |  |  |  |  |  |  |  |  |  |  |  | **X** |  |  |  |  |  |  |  |  |  | 0.1250 |
| **H10** |  |  |  |  |  |  |  |  |  |  |  |  |  |  |  | X | X | X |  |  |  |  |  |
| **H11** |  |  | X | X |  |  |  |  |  | X |  |  |  |  |  | X |  |  |  |  |  |  |  |
| **H12** | X | X | X | X | X | X | X | X | X | X |  | X | X | X | X | X | X | X |  | X |  |  |  |
| **H13** |  |  |  |  |  |  |  |  |  | X |  |  |  |  |  |  |  |  |  | X |  |  |  |
| **H14** |  |  |  |  |  |  |  | X |  |  |  | X |  |  | X |  |  | X |  |  |  |  |  |
| **H15** |  | X |  |  |  |  |  |  |  |  | X |  |  |  |  |  |  |  |  |  |  |  |  |
| **H16** |  | X |  |  |  |  |  |  |  |  | X |  |  |  |  |  |  | X |  | X |  |  |  |
| **H17** |  | X |  |  |  |  |  |  |  |  |  |  |  |  |  |  |  |  |  | X |  |  |  |
| **H18** |  |  |  |  |  |  |  |  |  |  |  |  |  |  | **X** |  |  |  |  |  |  |  | 0.0333 |
| **H19** |  |  |  |  |  |  |  | **X** |  |  |  |  |  |  |  |  |  |  |  |  |  |  | 0.1000 |
| **H20** |  | X |  |  |  |  | X | X |  |  |  |  | X |  | X |  |  | X |  |  |  |  |  |
| **H21** |  |  |  |  |  |  |  |  |  |  |  |  |  | **X** |  |  |  |  |  |  |  |  | 0.1429 |
| **H22** |  | X |  |  |  |  |  |  |  |  | X |  | X |  |  |  |  |  |  | X |  |  |  |
