# Supplementary material for: Population genetic structure and variability in Lindera glauca (Lauraceae) indicates low levels of genetic diversity and skewed sex ratios in natural populations in mainland China
Source: PeerJ. 2020 Jan 3;8:e8304. doi: 10.7717/peerj.8304 (PMC6944114; doi:10.7717/peerj.8304)
Supplement: Table S4 [file peerj-08-8304-s004.doc]

**Table S4.** **Pairwise geographic distances (km) above the diagonal and pairwise *F*_ST_ below the diagonal for 22 populations of *L. glauca*.**

|  | **ATM** | **JGS** | **LDZ** | **SJG** | **NTB** | **YTH** | **DBS** | **HMF** | **TMS** | **SQS** | **LYS** | **KYS** | **FJS** | **WYS** | **ZJS** | **WJS** | **GJS** | **NHS** | **FHS** | **ZJJ** | **SZY** | **HZY** |
| --- | --- | --- | --- | --- | --- | --- | --- | --- | --- | --- | --- | --- | --- | --- | --- | --- | --- | --- | --- | --- | --- | --- |
| **ATM** | — | 199.3 | 181.4 | 43.5 | 275.7 | 8.2 | 10.8 | 342.0 | 401.8 | 271.4 | 274.6 | 728.4 | 800.9 | 294.8 | 332.6 | 931.1 | 1033.3 | 393.2 | 824.6 | 602.8 | 591.4 | 464.3 |
| **JGS** | 0.046* | — | 19.1 | 161.7 | 76.8 | 201.2 | 198.5 | 193.7 | 601.0 | 465.3 | 466.8 | 894.8 | 613.2 | 476.8 | 526.9 | 734.5 | 839.7 | 253.8 | 626.1 | 415.1 | 789.4 | 396.6 |
| **LDZ** | 0.107 | 0.061 | — | 143.1 | 94.3 | 183.6 | 181.1 | 208.9 | 582.9 | 448.8 | 447.9 | 875.9 | 632.3 | 462.0 | 508.1 | 753.4 | 858.8 | 268.8 | 644.7 | 434.3 | 770.9 | 405.6 |
| **SJG** | 0.074 | 0.077 | 0.121 | — | 237.0 | 49.1 | 48.5 | 319.7 | 440.6 | 314.4 | 305.9 | 748.2 | 770.2 | 338.2 | 365.6 | 896.0 | 1000.0 | 374.0 | 787.7 | 571.8 | 627.7 | 462.5 |
| **NTB** | 0.099 | 0.109 | 0.176 | 0.065 | — | 277.8 | 275.2 | 176.3 | 677.2 | 541.9 | 540.3 | 957.9 | 547.3 | 552.2 | 600.9 | 661.5 | 769.0 | 234.1 | 551.3 | 351.0 | 864.6 | 403.6 |
| **YTH** | 0.126 | 0.130 | 0.194 | 0.069 | 0.025* | — | 3.9 | 339.7 | 400.3 | 267.4 | 275.8 | 731.9 | 800.2 | 289.2 | 333.3 | 931.7 | 1033.3 | 390.3 | 825.8 | 602.4 | 590.8 | 458.5 |
| **DBS** | 0.108 | 0.096 | 0.144 | 0.125 | 0.154 | 0.194 | — | 336.0 | 403.2 | 269.3 | 279.5 | 735.8 | 796.8 | 290.3 | 336.8 | 928.6 | 1030.0 | 386.5 | 822.8 | 598.9 | 593.9 | 454.7 |
| **HMF** | 0.145 | 0.122 | 0.137 | 0.150 | 0.173 | 0.221 | 0.071 | — | 722.2 | 564.0 | 614.8 | 1067.9 | 471.7 | 552.0 | 670.4 | 622.9 | 712.4 | 60.3 | 533.1 | 280.1 | 918.2 | 233.9 |
| **TMS** | 0.077 | 0.058 | 0.119 | 0.094 | 0.103 | 0.120 | 0.115 | 0.135 | — | 169.5 | 167.0 | 467.6 | 1192.5 | 219.0 | 117.0 | 1330.8 | 1429.7 | 762.9 | 1226.0 | 996.6 | 196.9 | 762.7 |
| **SQS** | 0.184 | 0.196 | 0.276 | 0.187 | 0.221 | 0.250 | 0.199 | 0.168 | 0.204 | — | 176.1 | 613.9 | 1035.7 | 69.9 | 184.7 | 1181.2 | 1275.6 | 600.8 | 1081.0 | 842.2 | 364.3 | 593.2 |
| **LYS** | 0.210 | 0.148 | 0.251 | 0.199 | 0.240 | 0.265 | 0.259 | 0.258 | 0.179 | 0.236 | — | 473.3 | 1075.2 | 245.8 | 61.6 | 1201.3 | 1305.9 | 663.1 | 1091.5 | 877.0 | 328.8 | 701.5 |
| **KYS** | 0.160 | 0.140 | 0.194 | 0.124 | 0.151 | 0.166 | 0.191 | 0.157 | 0.173 | 0.248 | 0.311 | — | 1505.1 | 677.2 | 435.2 | 1607.0 | 1720.8 | 1121.1 | 1488.9 | 1308.5 | 364.3 | 1174.3 |
| **FJS** | 0.253 | 0.212 | 0.233 | 0.199 | 0.273 | 0.267 | 0.257 | 0.273 | 0.254 | 0.271 | 0.258 | 0.324 | — | 1021.7 | 1133.5 | 184.6 | 244.0 | 439.8 | 180.4 | 198.3 | 1387.6 | 543.0 |
| **WYS** | 0.235 | 0.231 | 0.355 | 0.243 | 0.231 | 0.282 | 0.268 | 0.233 | 0.221 | 0.103 | 0.287 | 0.319 | 0.374 | — | 253.0 | 1174.1 | 1263.9 | 582.8 | 1078.5 | 832.0 | 404.5 | 554.3 |
| **ZJS** | 0.172 | 0.158 | 0.178 | 0.152 | 0.157 | 0.149 | 0.195 | 0.219 | 0.169 | 0.240 | 0.245 | 0.184 | 0.221 | 0.280 | — | 1261.5 | 1365.4 | 717.2 | 1152.2 | 935.5 | 267.2 | 745.8 |
| **WJS** | 0.204 | 0.147 | 0.173 | 0.186 | 0.181 | 0.194 | 0.166 | 0.204 | 0.153 | 0.288 | 0.238 | 0.248 | 0.236 | 0.374 | 0.165 | — | 123.0 | 601.1 | 122.4 | 343.6 | 1522.5 | 722.6 |
| **GJS** | 0.206 | 0.147 | 0.173 | 0.195 | 0.205 | 0.218 | 0.164 | 0.205 | 0.158 | 0.284 | 0.241 | 0.273 | 0.227 | 0.372 | 0.194 | 0.023* | — | 683.2 | 244.8 | 433.5 | 1623.4 | 782.1 |
| **NHS** | 0.191 | 0.160 | 0.200 | 0.181 | 0.189 | 0.204 | 0.145 | 0.191 | 0.144 | 0.228 | 0.194 | 0.263 | 0.219 | 0.253 | 0.187 | 0.046* | 0.053 | — | 520.1 | 258.4 | 959.5 | 191.4 |
| **FHS** | 0.351 | 0.343 | 0.427 | 0.295 | 0.230 | 0.251 | 0.412 | 0.391 | 0.307 | 0.347 | 0.358 | 0.317 | 0.372 | 0.369 | 0.289 | 0.358 | 0.389 | 0.309 | — | 264.3 | 1415.5 | 663.8 |
| **ZJJ** | 0.245 | 0.213 | 0.258 | 0.192 | 0.221 | 0.215 | 0.251 | 0.281 | 0.226 | 0.270 | 0.228 | 0.314 | 0.148 | 0.328 | 0.207 | 0.212 | 0.219 | 0.176 | 0.228 | — | 1190.9 | 402.5 |
| **SZY** | 0.578 | 0.594 | 0.622 | 0.522 | 0.464 | 0.482 | 0.663 | 0.623 | 0.555 | 0.623 | 0.642 | 0.616 | 0.549 | 0.668 | 0.409 | 0.550 | 0.586 | 0.535 | 0.477 | 0.500 | — | 956.3 |
| **HZY** | 0.491 | 0.483 | 0.561 | 0.460 | 0.377 | 0.389 | 0.530 | 0.474 | 0.420 | 0.538 | 0.578 | 0.509 | 0.526 | 0.598 | 0.392 | 0.454 | 0.492 | 0.418 | 0.396 | 0.454 | 0.267 | — |

*Notes*: * significant values with P ≤ 0.05.
